# Chicago Veterinary College

**Published:** 1891-07

**Authors:** 


*Chicago Veterinary College.*—A. S. Alexander, B. A., has been appointed to the Chair of Hygiene, vice Jonathan Periam.

A course of practical inspection of meat and milk, has been added to the curriculum.

Dr. Billings, late of the faculty, has received an appointment at the University of Nebraska.

